# CaMKII‐dependent ryanodine receptor phosphorylation mediates sepsis‐induced cardiomyocyte apoptosis

**DOI:** 10.1111/jcmm.15470

**Published:** 2020-07-24

**Authors:** Marisa Sepúlveda, Juan Ignacio Burgos, Alejandro Ciocci Pardo, Luisa González Arbelaez, Susana Mosca, Martin Vila Petroff

**Affiliations:** ^1^ Centro de Investigaciones Cardiovasculares Conicet La Plata Facultad de Ciencias Médicas Universidad Nacional de La Plata La Plata Argentina

**Keywords:** apoptosis, CaMKII, mitochondrial dysfunction, ryanodine receptors, Sepsis

## Abstract

Sepsis is associated with cardiac dysfunction, which is at least in part due to cardiomyocyte apoptosis. However, the underlying mechanisms are far from being understood. Using the colon ascendens stent peritonitis mouse model of sepsis (CASP), we examined the subcellular mechanisms that mediate sepsis‐induced apoptosis. Wild‐type (WT) CASP mice hearts showed an increase in apoptosis respect to WT‐Sham. CASP transgenic mice expressing a CaMKII inhibitory peptide (AC3‐I) were protected against sepsis‐induced apoptosis. Dantrolene, used to reduce ryanodine receptor (RyR) diastolic sarcoplasmic reticulum (SR) Ca^2+^ release, prevented apoptosis in WT‐CASP. To examine whether CaMKII‐dependent RyR2 phosphorylation mediates diastolic Ca^2+^ release and apoptosis in sepsis, we evaluated apoptosis in mutant mice hearts that have the CaMKII phosphorylation site of RyR2 (Serine 2814) mutated to Alanine (S2814A). S2814A CASP mice did not show increased apoptosis. Consistent with RyR2 phosphorylation‐dependent enhancement in diastolic SR Ca^2+^ release leading to mitochondrial Ca^2+^ overload, mitochondrial Ca^2+^ retention capacity was reduced in mitochondria isolated from WT‐CASP compared to Sham and this reduction was absent in mitochondria from CASP S2814A or dantrolene‐treated mice. We conclude that in sepsis, CaMKII‐dependent RyR2 phosphorylation results in diastolic Ca^2+^ release from SR which leads to mitochondrial Ca^2+^ overload and apoptosis.

## 1 INTRODUCTION

Experimental and clinical evidence indicates that cardiac contractile dysfunction is a complication that significantly enhances the mortality of sepsis.^1^, ^2^ Accumulating evidence demonstrates that cardiomyocyte apoptosis associated with mitochondrial damage plays an important role in the pathogenesis of sepsis‐induced contractile dysfunction.^3^, ^4^, ^5^, ^6^ Indeed, caspase‐3 inhibition has been shown to reduce apoptosis and improve cardiac contractile function in an endotoxin‐treated rat model of sepsis.^4^ However, the subcellular mechanisms underlying mitochondrial damage‐induced apoptosis in sepsis are not completely understood. Elucidating the mechanisms that mediate apoptosis may lead to the development of novel therapeutic targets and strategies for the management of sepsis‐induced cardiac dysfunction.

Ca^2+^‐calmodulin‐dependent protein kinase II (CaMKII) is a ubiquitous kinase that is canonically activated by the elevation of intracellular Ca^2+^ and more recently, its activity has also been shown to be modulated by ROS‐dependent oxidation.^7^, ^8^ CaMKII is known to play an important physiological role in the regulation of cardiac excitation‐contraction coupling.^9^ However, under pathological conditions CaMKII has been shown be detrimental.^10^, ^11^, ^12^ Indeed, we have recently shown that in sepsis, CaMKII is activated by oxidation and once active it phosphorylates the cardiac ryanodine receptor (RyR2) leading to inappropriate diastolic Ca^2+^ release from the sarcoplasmic reticulum (SR), known as SR Ca^2+^ leak. This SR Ca^2+^ leak reduces SR Ca^2+^ content and thus systolic Ca^2+^ release, mediating at least in part the contractile dysfunction associated with sepsis.^13^ In addition, CaMKII has been shown to be a common intermediate of diverse death stimuli that induce apoptosis of cardiac cells.^14^ Consistent with these results, we have previously demonstrated that CaMKII activation is a critical step in the signalling cascade that leads to apoptosis in ischaemia/reperfusion injury, ouabain toxicity, sustained Angiotensin II stimulation and rapid pacing.^8^, ^15^, ^16^, ^17^ Moreover, we and others have provided evidence demonstrating that CaMKII, through the phosphorylation of the RyR2, enhances SR Ca^2+^ leak which is then taken up by the mitochondria, leading to mitochondrial Ca^2+^ overload which triggers the apoptotic cascade.^17^, ^18^, ^19^, ^20^, ^21^, ^22^ The above‐mentioned events could also hold true for sepsis. Thus, we hypothesized that CaMKII‐dependent SR‐Ca^2+^ leak mediates apoptosis in sepsis.

The results presented herein, using a colon ascendant stent peritonitis (CASP) model of sepsis that closely mimics the clinical course of diffuse peritonitis with early and steadily increasing systemic infection and inflammation,^23^ enhanced apoptosis ^24^ and contractile dysfunction^13^ provide mechanistic insight on sepsis‐induced apoptosis. We show that in sepsis, cardiac cell death involves CaMKII activation, which through the phosphorylation of RyR2 enhances SR‐Ca^2+^ leak leading to mitochondrial Ca^2+^ overload and apoptosis. Importantly, we further show that the RyR2 plays a critical role in sepsis‐induced cell death and that agents that reduce RyR2 open probability (RyR2 stabilizers) are effective in preventing apoptosis, suggesting a potential therapeutic advantage of these compounds to reduce apoptosis and contractile dysfunction associated with sepsis.

## 2 MATERIALS AND METHODS

All procedures followed during this investigation were approved by Animal Welfare Committee of La Plata School of Medicine (University of La Plata), following the Guide for the Care and Use of Laboratory Animals published by the National Research Council, National Academy Press, Washington DC 2010 and/or European Union Directive for Animal Experiments 2010/63/EU. Three‐month‐old male wild‐type (WT) C57BL/6 mice and transgenic (TG) mice with cardiomyocyte‐delimited transgenic expression of either a CaMKII inhibitory peptide (AC3‐I) or a scrambled control peptide (AC3‐C) or mutant mice where the CaMKII‐dependent phosphorylation site on the RyR2 (site Serine 2814) is mutated to Alanine (S2814A) were used. All genetically modified mice were generated on a C57BL/6 background.

### 2.1 Mouse model of sepsis

Colon ascendens stent peritonitis (CASP) surgery was performed to induce sepsis as previously described.^13^ In brief, a small stent was inserted into the ascending colon of mice leading to the continuous leakage of intestinal bacteria into the peritoneal cavity resulting in peritonitis and systemic bacteraemia. Sham surgery was carried out using the identical surgical procedure, but without stent implantation. For more detail see online Supporting Information.

### 2.2 Cardiomyocyte isolation

Myocytes were isolated by enzymatic digestion.^13^ Details are provided under Supporting Information.

### 2.3 Isolation of mouse heart mitochondria

Isolated hearts were washed and homogenized in ice‐cold isolation solution (IS) consisting of 75 mmol/L sucrose, 225 mmol/L mannitol and 0.01 mmol/L EGTA neutralized with Trizma buffer at pH 7.4. After tissue pieces were settled, the entire supernatant was discarded and fresh IS was added and the mixture was transferred to a hand homogenizer. Proteinase (0.08 mg, bacterial, type XXIV, Sigma,) was added just before starting the homogenization procedure. The homogenate was carefully transferred after each step to a polycarbonate centrifuge tube. After 5 minutes of 750 × *g* of centrifugation to discard unbroken tissue and debris, the supernatant was centrifuged at 8000 × *g* for 10 minutes to sediment the mitochondria. The mitochondrial pellet was washed twice with IS and the last one with suspension solution (IS without EGTA, SS) at 8000 × *g* for 5 minutes each. The residue was washed and re‐suspended in SS. The mitochondrial protein concentration was evaluated by the Bradford method^25^ using bovine serum albumin as standard.

### 2.4 Apoptosis assessment

Mitochondrial Cytochrome C levels and the ratio between pro‐ and anti‐apoptotic proteins Bax and Bcl‐2, respectively, were used as an index of apoptosis (see online Supporting Information for details). Apoptosis was also determined by TUNEL assay (In Situ Cell Death Detection Kit, TMR red, Roche, Mannheim, Germany). TUNEL‐positive cells were imaged under a fluorescence microscope (100 × magnification) and counted in 10 random fields from each experimental situation. The results were expressed as percentage of TUNEL‐positive cells related to total number of cells. DAPI (1 µg/mL, 4', 6‐Diamidino‐2‐phenylindole dihydrochloride, Sigma, St. Louis, MO) was used for nuclear staining.

### 2.5 Immunoblotting

Homogenates, cytosolic fractions and SR membranes were prepared from the pulverized ventricular tissue. Proteins were electrophoresed and transferred to polyvinylidene fluoride membranes.^26^ Blots were probed with antibodies raised against, anti‐Bcl2 and anti‐Bax. Immunoreactivity was visualized by a peroxidase‐based chemiluminescence detection kit (Immobilon Western Millipore) using a Chemidoc Imaging System (Bio‐Rad, Hercules, CA). Details are provided in the Supporting Information.

### 2.6 Mitochondrial Membrane Potential

Mitochondrial membrane potential changes were evaluated by measuring rhodamine‐123 (RH‐123) fluorescence quenching in a buffer containing (in mM): 120 KCl, 20 MOPS, 10 Tris‐HCl and 5 KH2PO4 adjusted to pH = 7.4 containing RH‐123 0.1 μmol/L. Isolated mitochondria loaded with RH‐123 were excited at 503 nm, and fluorescence emission was detected at 527 nm.^27^ During the measurements, the reaction medium containing mitochondria (0.1 mg/mL) was continuously stirred. Mitochondrial membrane potential (ΔΨm) was calculated following the instructions previously detailed using the Nernst‐Guggenheim Equation^28^ According to the authors, RH‐123 uptake is in proportion to ΔΨm; therefore, the rate of fluorescence quenching is a function of ΔΨm, as well as the steady‐state level of fluorescence decrease.

### 2.7 Mitochondrial calcium retention capacity assay

The ability of mitochondria to retain exogenous Ca^2+^ before the irreversible opening of the mitochondrial permeability transition pore (mPTP) occurs was monitored by following the changes in the fluorescence of the Ca^2+^ sensitive indicator, Calcium Green‐5N.^29^ The reaction begins with the addition of successive pulses of 5 µmol/L Ca^2+^. After each addition, the fluorescence in the medium increases until the mitochondria start to take up Ca^2+^ and then it decreases. When mitochondria are sufficiently loaded with Ca^2+^, the opening of the mPTP occurs and the release of the mentioned ion in the medium increases again the fluorescence.^30^ For this assay, 0.3 mg/mL of isolated mitochondria were suspended in 2 mL buffer containing, 150 mM sucrose, 50 mM KCl, 2 mM KH2PO4 and 5 mM succinate in 20 mM LTris/HCl, (pH 7.4). At the end of the preincubation period (300 s), successive pulses of 5 μmol/L Ca2+ were added. Extramitochondrial Ca2+ concentration was recorded with 0.5 μM Calcium Green‐5N (Invitrogen, Carlsbad, CA, USA) with excitation and emission wavelengthsset at 506 and 532nm, respectively. All experiments were performed at 37°C and with continuous stirring.

Calcium retention capacity (CRC) was defined here as the amount of Ca^2+^ required to trigger a massive Ca^2+^ release by isolated cardiac mitochondria.^29^ CRC is used as an indicator of the resistance of the mPTP to opening after matrix Ca^2+^ accumulation and expressed as nmol CaCl_2_ per mg of mitochondrial proteins.

### 2.8 Statistical analysis

The normal distribution of the data was corroborated with the Kolmogorov‐Smirnov test; unpaired Student's *t* test, Mann‐Whitney test and one‐way ANOVA were applied accordingly using GraphPad Prism 5.0 (GraphPad Software, USA). The data are presented as means ± SEM. The level of significance was set at *P < *.05.

## 3 RESULTS

### 3.1 Apoptosis is enhanced in a polymicrobial model of sepsis

**Figure 1:**
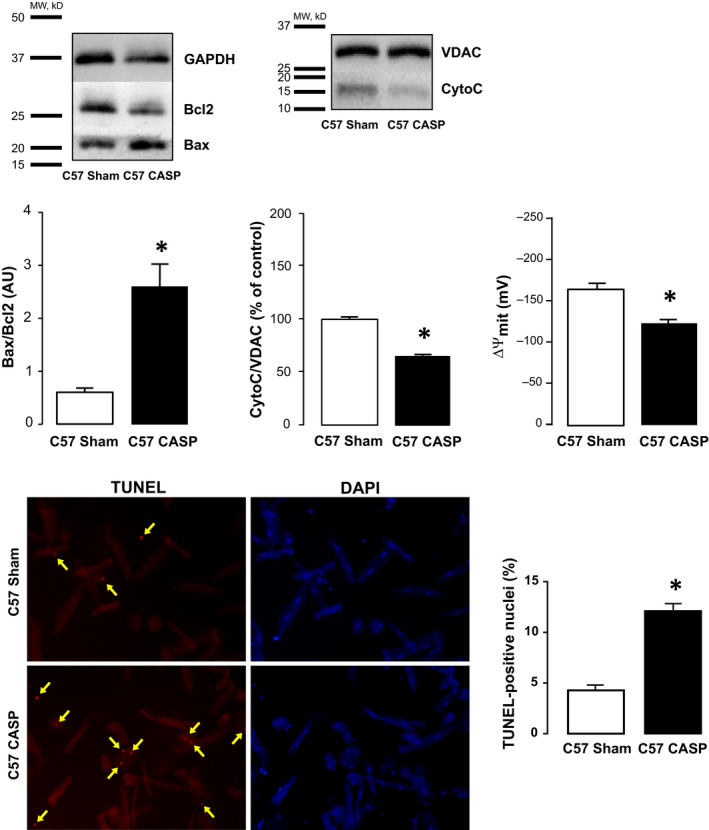
Polymicrobial model of sepsis is associated with apoptosis. Evaluation of activation of the apoptotic cascade. Representative blots and overall results showing that hearts of WT‐CASP mice have a significant increase the ratio between Bax and Bcl‐2 (Bax/Bcl2) relative to Sham controls (n = 4). WT‐CASP exhibited a significant decrease in the mitochondrial expression of cytochrome C (CytoC) compared to WT‐Sham mice (n = 4) (unpaired *t* test). Myocardial mitochondrial inner membrane potential (ΔΨm) changes were examined using rhodamine‐123 (RH‐123) fluorescence. As shown, ΔΨm measured in isolated mitochondria was significantly lower in WT‐CASP compared with WT‐Sham mice (n = 4) (*Mann‐Whitney* test). The bottom panel shows typical images and overall results of the increase in TUNEL‐positive cells in myocytes isolated from CASP mice compared to Sham myocytes. Scatter plot shows values from 3‐4 independent myocyte isolations (3‐4 hearts) *P* < .05 (unpaired *t* test). Data are expressed as means ± SEM * *P* < .05 vs Sham

Apoptosis was assessed by four independent methods, in heart homogenates, cardiomyocytes and cardiac mitochondria isolated from colon ascendens stent peritonitis operated mice (CASP) and from Sham‐operated controls. Heart homogenates, cardiomyocytes and cardiac mitochondria were prepared 24 hours after surgery when contractile dysfunction is already installed.^13^ Figure 1 depicts typical blots and overall results showing that the ratio between pro‐apoptotic (Bax) and anti‐apoptotic (Bcl2) proteins is increased in CASP homogenates relative to Sham, indicative of enhanced apoptosis in septic hearts. Similarly, mitochondrial cytochrome C content and mitochondrial membrane potential (ΔΨm) were significantly reduced in CASP vs Sham hearts showing that mitochondrial pathway‐induced apoptosis was enhanced in septic hearts. In addition, there was a significant increase in TUNEL‐positive nuclei in myocytes isolated from CASP compared to myocytes isolated from Sham mice.

### 3.2 CaMKII mediates apoptosis associated with sepsis

**Figure 2:**
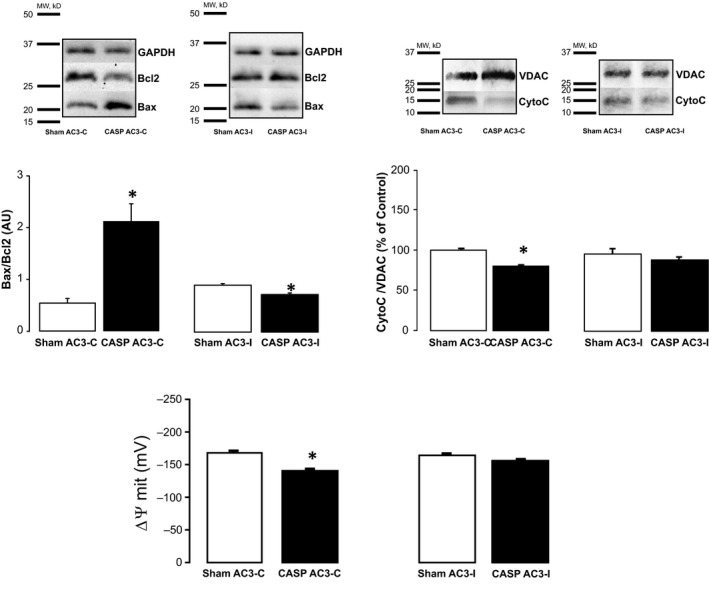
CaMKII mediates sepsis‐induced cardiac apoptosis. Representative immunoblots and overall results from heart homogenates and isolated mitochondria from CASP‐ and Sham‐operated transgenic mice expressing a CaMKII inhibitory peptide (AC3I) or mice expressing the scrambled control peptide (AC3C). CASP‐AC3C mice show a significant increase in Bax/Bcl2, a significant decrease in mitochondrial cytochrome C expression (unpaired *t* test) and a significant decrease in ∆ψm compared to Sham AC3C (n = 4) (*Mann‐Whitney* test). AC3I‐CASP mice did not show altered apoptotic indexes compared to mice to AC3I‐Sham mice (n = 4) indicating that CaMKII inhibition protects hearts from sepsis‐induced‐apoptosis. Data are expressed as means ± SEM * *P* < .05 vs Sham

We recently reported that CaMKII mediates at least part of the contractile dysfunction associated with sepsis, and several laboratories including our own have provided clear evidence that CaMKII is involved in triggering apoptosis in several disease models.^15^, ^16^, ^17^, ^20^, ^31^ To test whether CaMKII is also involved in sepsis‐induced apoptosis, we assessed apoptosis in heart homogenates and isolated mitochondria from CASP‐ and Sham‐operated transgenic mice, expressing either a CaMKII inhibitory peptide, AC3‐I, or a scramble non‐inhibitory peptide, AC3‐C. Figure 2 depicts typical blots and overall results of apoptotic indexes showing that apoptosis is significantly enhanced in hearts of CASP AC3‐C mice vs Sham, whereas hearts from AC3‐I mice are resistant to sepsis‐induced apoptosis.

### 3.3 Mechanisms underlying CaMKII‐dependent apoptosis in sepsi

**Figure 3:**
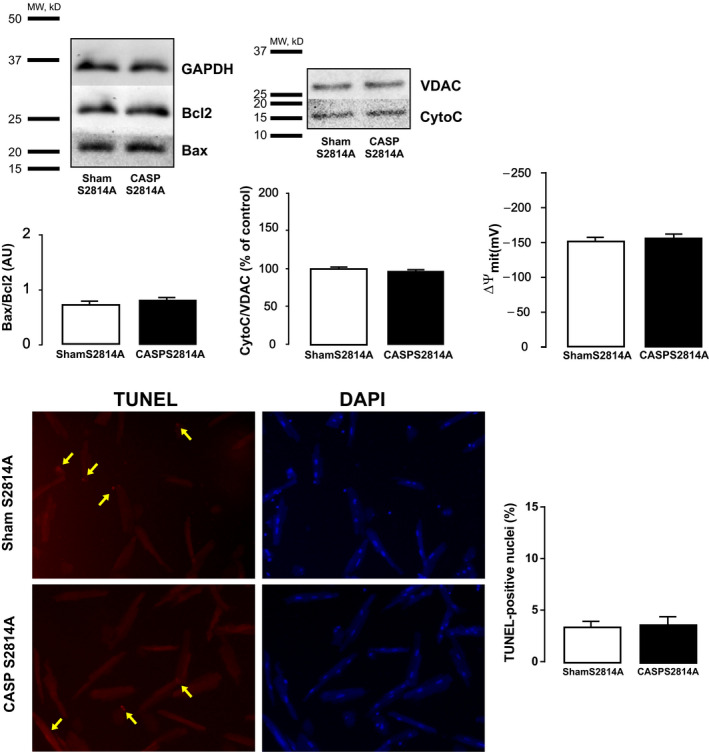
Preventing RyR2 phosphorylation protects against apoptosis induced by sepsis in mice. Typical Western blots, images and overall results showing that CASP‐operated S2814A mice do not show significant alterations in the apoptotic indexes Bax/Bcl2, mitochondrial cytochrome C expression, ∆ψm or TUNEL staining (n = 4 per group) compared to Sham‐operated S2814A mice. All data are expressed as means ± SEM * *P* < .05 (unpaired *t* test) vs Sham)

**Figure 4:**
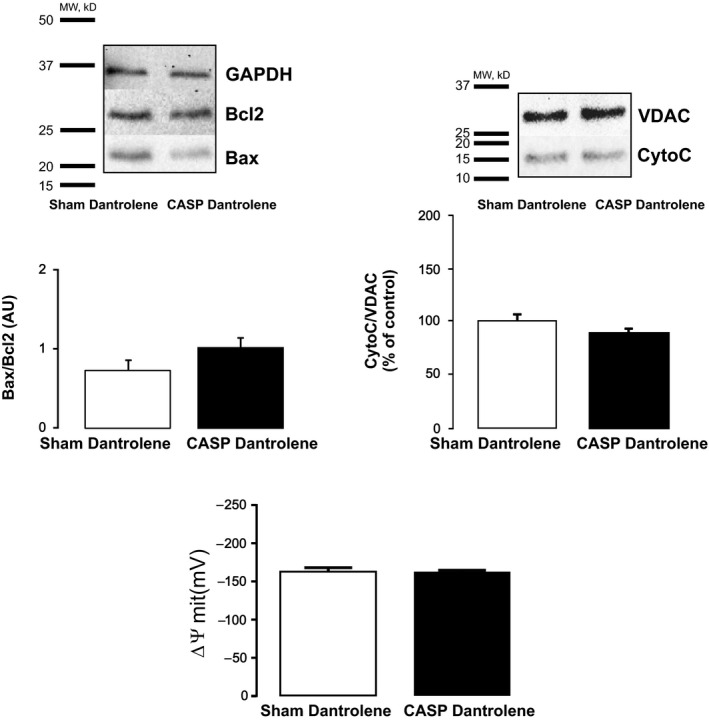
Reducing RyR2 open probability prevents sepsis‐induced‐apoptosis. Typical blots and overall results of the effect of dantrolene pretreatment on sepsis‐induced apoptosis. WT‐CASP + dantrolene mice hearts showed non‐significant (NS) changes in Bax/Bcl2, mitochondrial cytochrome C expression and ∆ψm compared to Sham + dantrolene‐operated WT mice (n = 4 per group). All data are expressed as means ± SEM * *P* < .05 (unpaired *t* test) vs Sham)

**Figure 5:**
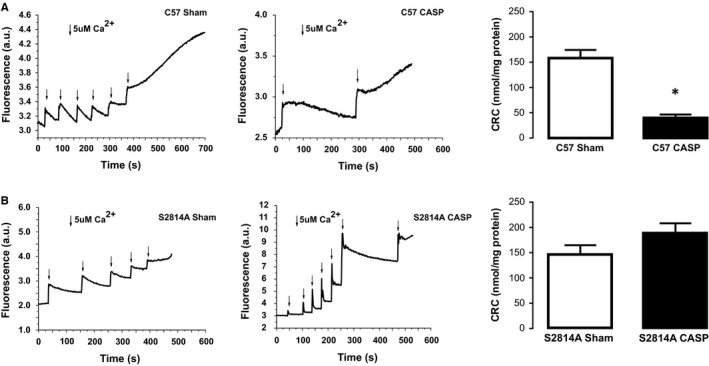
Role of mitochondrial Ca^2+^ in sepsis. A, Typical tracings of changes in the fluorescence of Ca^2+^ indicator Calcium Green‐5N. Arrows indicate Ca^2+^ addition to the mitochondrial suspension. Increasing extramitochondrial Ca^2+^ results in a rapid increase in the fluorescent signal followed by a decline in the fluorescence intensity of the sensor resulting from mitochondrial Ca^2+^ uptake. WT‐Sham mitochondria showed enhanced tolerance to extramitochondrial Ca^2+^ addition compared to WT‐CASP mitochondria. The measurement of extramitochondrial Ca^2+^ shows that WT‐Sham cardiac mitochondria are able to accumulate significantly more Ca^2+^ than WT‐CASP mitochondria (CRC) (n = 4). *Mann‐Whitney* test results are expressed as mean ± SEM. **B:** Typical traces and overall results of changes of Calcium Green fluorescence after Ca^2+^ addition in samples of mitochondria from S2814A mutant CASP mice CASP mice compared to Sham. Mitochondria from CASP mice showed preserved CRC compared to Sham (n = 4). Unpaired *t* test, results are expressed as mean ± SEM

RyR2 is crucial for physiological myocyte intracellular Ca^2+^ handling. Nevertheless, abnormal Ca^2+^ handling and Ca^2+^ leak via the RyR2 have recently been shown by us and others to be key determinants of cardiomyocyte apoptosis under different pathological conditions.^20^, ^32^, ^33^ Importantly, we recently showed that, in sepsis, CaMKII‐dependent RyR2 phosphorylation at site Serine 2814 results in enhanced SR Ca^2+^ leak favouring contractile dysfunction.^13^ Thus, using transgenic mice lacking the RyR2 CaMKII‐dependent phosphorylation site, Serine 2814 (S2814A), we examined the role of this phosphorylation on sepsis‐induced apoptosis. Figure 3 shows typical blots and overall data indicating that, similar to hearts from AC3‐I CASP mice, S2814A‐CASP mice are protected against sepsis‐induced apoptosis. In addition, when apoptosis was analysed by TUNEL staining, there was no difference between myocytes isolated form S2814A Sham and S2814A CASP mice. We further tested the involvement of RyR2 in sepsis‐induced cell death by using dantrolene, a pharmacological compound known to reduce RyR2 open probability.^34^ Figure 4 depicts typical blots and average results showing that 7‐day treatment of mice with an intraperitoneal injection (IP) of the RyR2 stabilizer, dantrolene (20 mg/kg body weight), was able to prevent sepsis‐induced apoptosis (Details are provided in the Supporting Information). Taken together, these results suggest that CaMKII‐dependent RyR2 phosphorylation, possibly through the enhancement of SR Ca^2+^ leak, is functionally involved in sepsis‐induced cell death. Enhanced Ca^2+^ leak from the SR has been shown to promote mitochondrial Ca^2+^ overload, resulting in the opening of the mitochondrial permeability transition pore (mPTP) and activation of the apoptotic cascade.^17^, ^20^, ^35^ To assess whether this is the mechanism underlying sepsis‐induced cell death, we measured the Ca^2+^ retaining capacity (CRC) of mitochondria isolated from Sham and CASP wild‐type mice. For this purpose, we performed experiments using Calcium Green‐5N to measure extramitochondrial Ca^2+^ levels (Figure 5). In these experiments, 5 µmol/L Ca^2+^ addition to mitochondria isolated from wild‐type Sham‐operated mice resulted in a rapid increase in Calcium Green fluorescence followed by a decline in the fluorescence intensity of the sensor. This slower decrease of fluorescence after Ca^2+^ addition is consistent with mitochondrial Ca^2+^ uptake.^36^ After sequential administration of 5 µmol/L pulses of Ca^2+^, mitochondrial CRC is exceeded and stored mitochondrial Ca^2+^ is released into the extramitochondrial space through the opening of the mPTP. As observed in the typical traces shown in Figure 5A, mitochondria isolated from CASP‐operated C57 mice resisted the addition of significantly fewer 5 µmol/L pulses of Ca^2+^ before the opening of the mPTP, suggesting that these mitochondria have higher resting intramitochondrial Ca^2+^ than Sham. In addition, Figure 5B depicts typical tracings and overall results of mitochondrial CRC showing that mitochondria isolated from S2814A mutant CASP mice have preserved CRC compared to Sham. Similar results were obtained in dantrolene‐pretreated CASP and Sham mice (Figure S1), confirming that mitochondria isolated from CASP mice are overloaded with Ca^2+^ resulting from CaMKII‐dependent RyR2 Ca^2+^ leak.

## 4 DISCUSSION

Our previous study demonstrates that CaMKII‐dependent SR‐Ca^2+^ leak reduces the cytosolic Ca^2+^ transient amplitude, contributing to the reduced contractility associated with sepsis.^13^ In addition, several reports have proposed myocyte cell death by apoptosis as a critical process that contributes to contractile dysfunction of the septic heart.^37^, ^38^, ^39^ Interestingly, sustained activation of CaMKII has been shown to be pro‐apoptotic.^10^ Indeed, experimental evidence from our laboratory demonstrates that CaMKII activation is a primary event in the signalling cascade that leads to apoptosis in ischaemia/reperfusion injury, ouabain toxicity, rapid pacing and under sustained Angiotensin II stimulation.^8^, ^15^, ^16^, ^17^ These results suggest that CaMKII, in addition to mediating a reduction in myocyte Ca^2+^ transient amplitude in sepsis, could favour the apoptotic process. However, the subcellular mechanisms underlying CaMKII‐dependent apoptosis in sepsis have yet to be determined. Thus, in the present study, using a colon ascendens stent peritonitis (CASP) mouse model that closely mimics the clinical situation of abdominal sepsis, we investigated the mechanisms involved in CaMKII‐dependent apoptosis.

Consistent with previous reports, we confirmed that the CASP model of sepsis promotes cardiac apoptosis, as evidenced by an enhanced ratio between pro‐ and anti‐apoptotic proteins, Bax and Bcl2, respectively (Bax/Bcl2), a decrease in mitochondrial cytochrome C levels, a significant decrease in mitochondrial membrane potential and by an increase in TUNEL staining. These results showing altered mitochondrial homeostasis further suggest that cardiac myocyte cell death, in sepsis, is mediated at least in part, by the mitochondrial pathway of apoptosis.

### 4.1 CaMKII mediates sepsis‐induced cell death

Oxidative stress has been shown to be enhanced in sepsis, and experimental evidence demonstrates that antioxidants can prevent cardiac dysfunction associated with sepsis.^40^, ^41^ Interestingly, we recently showed that oxidative activation of CaMKII leads to the phosphorylation of RyR2 site Serine 2814, promoting SR Ca^2+^ leak which in turn reduces SR Ca^2+^ content and contractility in the CASP model of sepsis.^13^ In addition, activation of CaMKII has been shown to be a common intermediate of diverse death stimuli that induce apoptosis in cardiac cells.^14^ Taken together, these results suggest that oxidative activation of CaMKII could underlie sepsis‐induced apoptosis. The results presented herein using a transgenic mouse model expressing a CaMKII inhibitory peptide (AC3‐I) show that CaMKII mediates sepsis‐induced cardiomyocyte apoptosis. Interestingly and consistent with our previously published results,^13^ CASP‐operated AC3‐I mice had a higher survival rate than CASP‐AC3‐C mice (see results in Supporting Information). To our knowledge, these are the first results showing that CaMKII inhibition can prevent sepsis‐induced apoptosis.

### 4.2 Mechanism underlying CaMKII‐dependent apoptosis in sepsis

Recent experimental evidence from our laboratory, in the setting of ischaemia and reperfusion injury, suggests that CaMKII may promote apoptosis by altering the coupling between SR Ca^2+^ release and mitochondrial Ca^2+^ uptake, resulting in mitochondrial Ca^2+^ overload and opening of the mPTP.^20^ Indeed, the interplay between SR and mitochondria under different stimuli has been known for many years to be pivotal in triggering apoptotic signals.^18^, ^19^ Supporting the critical role played by the SR/mitochondrial interaction in CaMKII‐dependent apoptosis, Zhang et al concluded that enhanced activity of CaMKII results in RyR2 phosphorylation leading to enhanced SR Ca^2+^ leak and mitochondrial Ca^2+^ elevation, associated with exacerbated cell death in transgenic mice lacking phospholamban and overexpressing CaMKIIαδc.^33^ Thus, we hypothesized that sepsis‐induced cell death could be, at least in part, due to CaMKII‐dependent post‐translational modification of the RyR2 resulting in SR Ca^2+^ leak which would be taken up by the mitochondria provoking mitochondrial Ca^2+^ overload. To test this hypothesis, we used myocytes isolated from transgenic mice with RyR2 site Serine 2814 mutated to Alanine (S2814A) and therefore not phosphorylatable by CaMKII. We observed that S2814A CASP mice were protected from sepsis‐induced apoptosis (Figure 3). Moreover, CASP‐operated S2814A mice had a higher survival rate than CASP‐operated WT mice (see results in Supporting Information). We have previously shown that CASP S2814A mice have a similar degree of CaMKII activation than CASP wild‐type mice. Moreover, site Thr17 of phospholamban, a specific target of CaMKII, also has a similar level of phosphorylation in CASP wild‐type mice and in CASP Ser2814A mice, confirming that CASP wild‐type and CASP Ser2814A mice have a similar level of CaMKII activation.^13^ Thus, Ser2814A mice are protected from apoptosis due to the absence of CaMKII‐dependent RyR2 phosphorylation in spite of CaMKII being able to phosphorylate other targets. These results suggest that in sepsis, CaMKII‐dependent RyR2 phosphorylation is critical for triggering apoptosis. Recent evidence suggests that CaMKII can also directly regulate mitochondrial Ca^2+^ uptake by the uniporter, leading to mitochondrial Ca^2+^ overload and mPTP opening.^21^ Although we cannot exclude the mitochondrial uniporter as an additional target for CaMKII contributing to enhanced apoptosis in sepsis, our results showing that hearts from S2814A CASP mice are protected from apoptosis suggest that CaMKII‐dependent uniporter phosphorylation is not a prerequisite for sepsis‐induced apoptosis.

High‐resolution Cryo‐EM studies have allowed the building of an atomic level model of the RyR channel core and cytoplasmic shell, permitting a better understanding of channel structure and function.^42^ The emerging model is that the RyR is a large complex of interconnecting helices which enable allosteric coupling and conformational changes between the shell and the pore of the channel that facilitate a range of stimuli to exert control over channel function.^43^ Dantrolene is a therapeutic agent used to treat malignant hyperthermia, and experimental studies have shown that it also protects against heart failure and arrhythmias by inhibiting Ca^2+^ release from the SR. Although the mechanism of action of dantrolene is not completely understood, Ye Win Oo et al showed that dantrolene inhibits RyRs by destabilizing their open state and stabilizing their closed state. Several studies provide evidence that dantrolene modulates interdomain interactions and that calmodulin binding to the RyR is required to produce dantrolene‐induced channel inhibition.^44^, ^45^, ^46^ Interestingly, a recent report has shown that dantrolene preserves cardiac mitochondria and prevents contractile dysfunction associated with sepsis.^34^ However, if this compound can also prevent sepsis‐induced apoptosis has not been evaluated. Consistent with this possibility, we recently reported that rapid pacing‐induced apoptosis, which is also CaMKII dependent, can be prevented by reducing RyR2 open probability with the carvedilol‐non‐β‐blocking analog, VK‐II‐86.^20^ Thus, we hypothesized that reducing RyR2 open probability with dantrolene could be effective preventing sepsis‐induced cell death. We show herein that dantrolene treatment is able to prevent sepsis‐induced cell death (Figure 4) and that this treatment also enhances survival rate of CASP‐operated WT mice (see results in online Supporting Information). These results highlight the possibility of using RyR2 stabilizers as a therapeutic strategy to reduce the adverse remodelling associated sepsis.

To assess whether CaMKII‐dependent increase is SR Ca^2+^ leak results in mitochondrial Ca^2+^ overload, we measured mitochondrial CRC in mitochondria isolated from Sham and CASP mice. As observed in Figure 5A, CRC was significantly reduced in wild‐type CASP mitochondria, suggesting that these mitochondria were Ca^2+^ overloaded and therefore tolerated less Ca^2+^ loading until mPTP opening compared to Sham. Consistent with mitochondrial Ca^2+^ overload resulting from CaMKII‐dependent RyR2 phosphorylation and enhanced Ca^2+^ leak from the SR, the decrease in CRC observed in CASP mitochondria was absent in CASP mitochondria isolated from S2814A mice (Figure 5B) and from mice treated with dantrolene (Figure S1). Taken together, and similar to the conclusion of Zhang et al^33^ our results suggest that sepsis‐induced CaMKII activation leads, at least in part, to an increase in RyR2 open probability and Ca^2+^ leak from the SR, which would result in mitochondrial Ca^2+^ overload, mPTP opening and cytochrome C release which would trigger the apoptotic cascade.

Limitations: Our results using dantrolene suggest that reducing RyR2 open probability can protect the heart from sepsis‐induced apoptosis and improve survival. However, dantrolene also targets RyR1 which has been shown to be localized in mitochondria.^47^,^48^ Thus, the protective effect of dantrolene could, at least in part, be due to its impact on RyR1. Our results using transgenic mice which have a specific point mutation of RyR2 site Serine 2814 mutated to Alanine show that these mice are protected from sepsis‐induced apoptosis providing solid evidence that RyR2 is the RyR isoform involved in sepsis‐induced apoptosis. Supporting this contention, we did not observe significant differences in mitochondrial Ca^2+^ uptake between Sham and Sham dantrolene‐treated WT mice suggesting that mitochondrial RyR1 inhibition is not involved in the anti‐apoptotic effects of dantrolene (Figure S2). Based on these observations, it is likely that the protective effect of dantrolene is mainly due to its effect on RyR2. Another potential limitation of our study is that we did not examine the mechanism underlying the enhanced survival of dantrolene‐treated mice. However, considering our previous results showing that CASP S2814A mice have preserved ejection fraction, SR Ca^2+^ content, Ca^2+^ transient amplitude and enhanced survival^13^ and those of a recent report demonstrating that dantrolene improves mitochondrial and contractile function in a model of sepsis induced by LPS administration,^34^ it is likely that the improvement in survival of dantrolene‐treated CASP mice is due to its ability to reduce SR diastolic Ca^2+^ release and ameliorate Ca^2+^ handling and mitochondrial function in CASP mice. Future experiments designed to assess Ca^2+^ handling in dantrolene‐treated CASP and Sham mice would be important to directly answer these limitations.

In summary, our results show for the first time that CaMKII plays a pivotal role in sepsis‐induced apoptosis. In addition, our results shed mechanistic insight indicating that CaMKII‐dependent RyR2 phosphorylation promotes SR Ca^2+^ leak that results in mitochondrial Ca^2+^ overload which triggers apoptosis. More importantly, our results showing that stabilizing the RyR2 with dantrolene can prevent sepsis‐induced cell death suggests the potential use of this agent for the treatment of the adverse remodelling associated with sepsis. Taken together, these findings propose CaMKII and RyR2 as new therapeutic targets whose inhibition or stabilization, respectively, could serve to ameliorate the cardiac symptoms of sepsis.

## CONFLICT OF INTERESTS

The authors declare that they have no conflict of interest.

## AUTHOR CONTRIBUTION

**Marisa Sepúlveda:** Formal analysis (equal); Investigation (lead); Methodology (equal); Writing‐original draft (lead). **Juan Ignacio Burgos:** Formal analysis (equal); Investigation (equal); Methodology (equal). **Alejandro Ciocci Pardo:** Formal analysis (equal); Investigation (equal); Methodology (equal). **Luisa González Arbelaez:** Formal analysis (equal); Investigation (equal); Methodology (equal). **Susana Mosca:** Investigation (equal); Supervision (equal). **Martín Vila Petroff:** Funding acquisition (lead); Investigation (lead); Writing‐original draft (lead); Writing‐review & editing (lead).

## Supporting information

Supplementary Material

## Data Availability

The data used to support the findings of this study are available from the corresponding author upon request.
