# Supplementary material for: CaMKII‐dependent ryanodine receptor phosphorylation mediates sepsis‐induced cardiomyocyte apoptosis
Source: J Cell Mol Med. 2020 Jul 24;24(17):9627–37. doi: 10.1111/jcmm.15470 (PMC7520277; doi:10.1111/jcmm.15470)
Supplement: Supplementary file 1 — Supplementary Material [file JCMM-24-9627-s001.docx]

Supplemental material:

*Mouse model of sepsis*

Colon ascendens stent peritonitis (CASP) is reproducible model that closely mimics the clinical situation of abdominal sepsis. To obtain the CASP model the animals wereanaesthetised with an intraperitoneal injection of ketamine/midazolam 100mg/kg + 5mg/kg. After disinfecting the abdominal skin a small midline incision was made to open the abdominal wall. A 14-gauge stent (2.0x45mm, 270ml/min) was inserted into the ascending part of colon, approximately 15mm above the ileocaecal valve and fixed with a 7/0 suture (Tagum black, non absorbable). To ensure safe intraluminal passage the stent was filled with a small amount of feces. The caecum was relocated into the abdominal cavity and the abdominal wall and skin were sutured with a 5/0 suture (Tagum black, non absorbable).

Sham surgery was carried out using the identical surgical procedures, but without stent implantation. After surgery, mice received an intraperitoneal administration of a powerful analgesic (Tramadol 0.1 mg/kg bodyweight (BW)). Finally, fluid resuscitation was attained by intraperitoneal administration of 50ml/kg BW of a 0.9% saline solution.

24h after surgery, echocardiographic recordings were performed and subsequently mice were sacrificed by intraperitoneal injection of sodium pentobarbital (45mg/kg) and hearts were excised when plane three of phase III of anaesthesia was reached. Plane three of phase III of anaesthesia was verified by the presence of slow deep diaphragmatic breathing, loss of the corneal reflex and the absence of tongue retraction.

*Administration of Dantrolene*

The C57BL-6 mice were treated with Dantrolene sodium 20 mg/kg body weight (1,2), which was purchased from Sigma-Aldrich (St. Louis, MO, USA) by IP injection during seven days. After treatment, the mice were divided into 2 groups: 1) Dantrolene Sham and 2) Dantrolene CASP, surgery was performed and 24 hours later were sacrificed. The mice were monitored for survival.

*Cardiomyocyte isolation*

Sham and CASP-operated WT and TG mice were anesthetized by intra-abdominal injection of ketamine/midazolam (100 mg/kg + 5mg/kg BW). Myocytes were isolated by enzymatic digestion (3) and kept, until use, at room temperature (20–22°C) in a 2-hydroxyethyl)-1-piperazineethanesulfonic acid (HEPES) buffered solution, containing (in mM): 146.2 NaCl, 4.7 KCl, 1.8 CaCl2, 10.0 HEPES, 0.4 NaH2PO4, 1.1 MgCl2, 10 glucose (pH was adjusted to 7.4 with NaOH).

*Western Blotting*

*Preparation of hearts*

Animals were sacrificed by cervical dislocation and hearts were immediately removed via a midline sternotomy. Beating hearts were transferred into cold PBS and allowed to beat spontaneously until no more blood was pumped out of the aortic root. They were then snap frozen in liquid nitrogen and stored at -80°C for further use.

*Gel electrophoresis and immunoblot analysis*

SDS-Page was performed in a criterion gel system (Bio-Rad). The resolving gels were on 6% or 10%.Together with the samples, a molecular weight marker (Precision Plus Protein^TM^ Dual Color Standards#1610374, BIO-RAD) was seeded in order to then recognize the weight of the protein fraction of interest. The gels were run al 200V constant voltage for ~1 hour in standard Laemmli running buffer.After the gels finished running, they were immediately placed in transfer buffer to equilibrate. The gels were transferred onto 0.2 micron PVDF membrane (Inmobilon Millipore) at 60V constant voltage for 1 h. Blots were blocked overnight at 4°C in 5% nonfat dry milk diluted in TBST (50mM Tris-base, 200mM NaCl, and 0.05% Tween-20, pH 7.5. Primary monoclonal antibodies dilutions antibody were used to detect anti-Bcl2 (1:1000, Abcam,#ab59348, EE.UU) and anti-Bax (1:1000, Abcam, #ab53154, EE.UU). The GAPDH signal was used as a loading control(1:500, Invitrogen, #PA1-988, EE.UU).Primary antibodies were incubated at 4°C overnight. The blots were then washed and incubated in secondary antibody (goat anti-rabbit#sc-2004 and goat anti-mouse#sc-516102, Santa Cruz Biotechnology, EE.UU) for 2 h at room temperature. The secondary antibody was diluted 1:20000 in the same dilution buffer as the primary. After being washed, blot immunoreactivity was visualized by a peroxidase-based chemiluminescence detection kit (Immobilon Western Millipore) using a Chemidoc Imaging System. The signal intensity of the bands was quantified using ImageJ (NIH).

*Determination of the mitochondrial cytochrome C*

The LV was homogenized and a mitochondrial fraction was isolated. Briefly, LV were homogenized in ice-cold RIPA buffer (300mmol L−1 sucrose, 1mmol L−1 DTT, 4mmol L−1EGTA, 20mmol L−1 Tris, pH 7.4, 1% Triton X, 10% protease cocktail, 25μmol L−1 FNa, 1μmol L−1 orthovanadate). From the supernatant, proteins (100 μg) were resolved by SDS-PAGE and transferred to PVDF membranes (2 h).Molecular weight marker (Precision Plus Protein^TM^All Blue Prestained Protein Standards #1610373, BIO-RAD) was seeded in order to then recognize the weight of the protein fraction of interest. The membranes were blocked with 5% non-fat milk in Tris-buffered saline (pH 7.5) containing 0.1% Tween (TBS-T), and probed overnight at 4 °C with antibodies against phosphorylated Cytochrome C (1:1000,#338500, Invitogen, EE.UU). The membranes were washed four times for 10 min with TBS-T prior to the addition of the anti-mouse secondary antibody (1:5000, #sc-516102, Santa Cruz Biotechnology, EE.UU) and protein bands were analysed by using a chemiluminescence system (ECL Plus; GE Healthcare Life Sciences). The mitochondrial voltage-dependent anion channel (VDAC, 1:1000, #V2139,Sigma-Aldrich , EE.UU) signal was used as a loading control.

Supplemental Results:

**Figure 1S. Reducing RyR2 open probability prevents mitochondrial dysfunction associated with sepsis.** Typical traces and overall results of changes of calcium green fluorescence after Ca^2+^ addition in samples of mitochondria from dantrolene pretreated WT CASP mice compared to Sham. Mitochondria from dantrolene pretreated CASP mice showed preserved CRC compared to Sham (n=4). There are no significant differences between groups Sham Dantrolene vs CASP Dantrolene. Results are expressed as mean ± SEM, statistical test: unpaired Student’s t test.

**
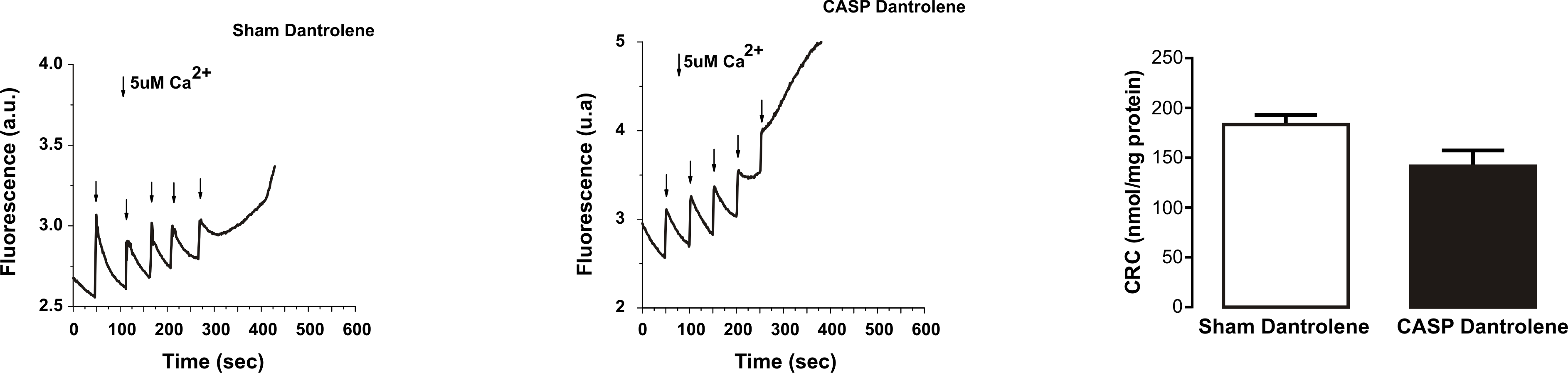
**

**Figure 2S. RyR1 isoform is not involved in the antiapoptotic effects of dantrolene in sepsis.** Considering that RyR1 is a mitochondrial Ca^2+^ uptake mechanism in heart that has been shown to be sensitive to dantrolene we examined the impact of this compound on mitochondrial Ca2+ dynamics. The time per pulse and Tau were measured from the CRC records of Sham and Sham-dantrolene-treated mice. The time per pulse (in sec), namely, the time it takes fluorescence to decrease after the increase caused by the addition of Ca^2+^, and the exponential decay constant (Tau, in sec) of mitochondrial Ca^2+^ uptake from fluorescence changes were measured and calculated. Tau was calculated according to the following equation: Ft = F0 e-t/Tau, in which Ft = fluorescence at t time, t = time, Tau = is the time at which F reaches 37% of initial F. These parameters were not significantly different between both groups (n=4 for each), suggesting that dantrolene does not affect mitochondrial Ca2+ handling under our experimental conditions. Results are expressed as mean ± SEM, statistical test: unpaired Student’s t test.


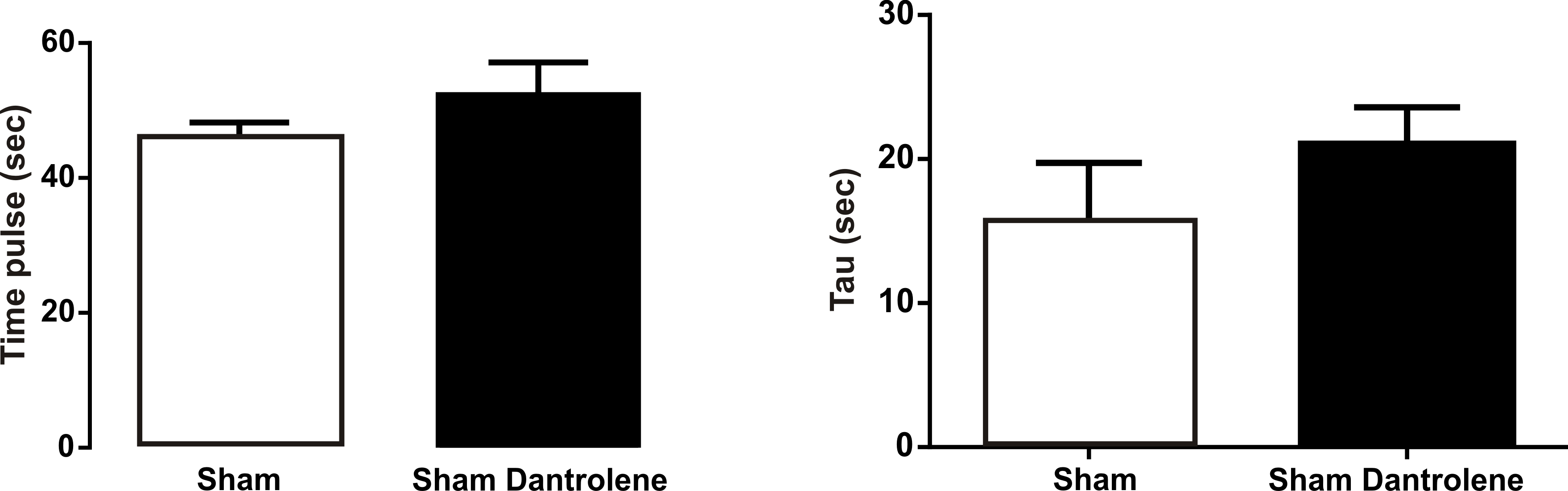


Animal survival post-surgery:

Consistent with previous results (4), mice subjected to CASP surgery using a 14G stent had 50% survival 24 hours after surgery. Similarly, we observed that CASP AC3-C mice had a 61.1% survival 24 hours after surgery. In contrast, CASP AC3-I mice had a higher survival rate 78.6%. Consistent with CaMKII-dependent RyR2 phosphorylation having a causal role in sepsis-induced apoptosis and contractile dysfunction, 83.3% mice that have the RyR2 CaMKII phosphorylation site Ser2814 mutated to alanine (Ser2814A) survived 24 hours after CASP surgery. Similarly, 84.6% mice treated with dantrolene survived 24 hours after CASP surgery. These results suggest that CaMKII inhibition, prevention of CaMKII-dependent RyR2 phosphorylation or a reduction in RyR2 open probability are able to improve survival of septic mice, at least in part by a reduction in apoptosis. The table below indicates the total number of mice operated and their survival 24 hours post-surgery.

Table 1:


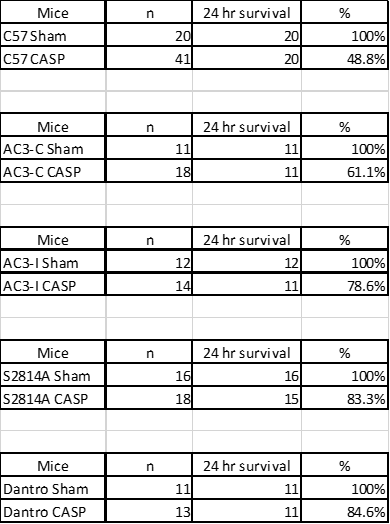


References:

1) [Kobayashi S](https://www.ncbi.nlm.nih.gov/pubmed/?term=Kobayashi%20S%5BAuthor%5D&cauthor=true&cauthor_uid=20944434), [Yano M](https://www.ncbi.nlm.nih.gov/pubmed/?term=Yano%20M%5BAuthor%5D&cauthor=true&cauthor_uid=20944434), [Uchinoumi H](https://www.ncbi.nlm.nih.gov/pubmed/?term=Uchinoumi%20H%5BAuthor%5D&cauthor=true&cauthor_uid=20944434), [Suetomi T](https://www.ncbi.nlm.nih.gov/pubmed/?term=Suetomi%20T%5BAuthor%5D&cauthor=true&cauthor_uid=20944434), [Susa T](https://www.ncbi.nlm.nih.gov/pubmed/?term=Susa%20T%5BAuthor%5D&cauthor=true&cauthor_uid=20944434), [Ono M](https://www.ncbi.nlm.nih.gov/pubmed/?term=Ono%20M%5BAuthor%5D&cauthor=true&cauthor_uid=20944434), [Xu X](https://www.ncbi.nlm.nih.gov/pubmed/?term=Xu%20X%5BAuthor%5D&cauthor=true&cauthor_uid=20944434), [Tateishi H](https://www.ncbi.nlm.nih.gov/pubmed/?term=Tateishi%20H%5BAuthor%5D&cauthor=true&cauthor_uid=20944434), [Oda T](https://www.ncbi.nlm.nih.gov/pubmed/?term=Oda%20T%5BAuthor%5D&cauthor=true&cauthor_uid=20944434), [Okuda S](https://www.ncbi.nlm.nih.gov/pubmed/?term=Okuda%20S%5BAuthor%5D&cauthor=true&cauthor_uid=20944434), [Doi M](https://www.ncbi.nlm.nih.gov/pubmed/?term=Doi%20M%5BAuthor%5D&cauthor=true&cauthor_uid=20944434), [Yamamoto T](https://www.ncbi.nlm.nih.gov/pubmed/?term=Yamamoto%20T%5BAuthor%5D&cauthor=true&cauthor_uid=20944434), [Matsuzaki M](https://www.ncbi.nlm.nih.gov/pubmed/?term=Matsuzaki%20M%5BAuthor%5D&cauthor=true&cauthor_uid=20944434). Dantrolene, a therapeutic agent for malignant hyperthermia, inhibits catecholaminergicpolymorphic ventricular tachycardia in a RyR2(R2474S/+) knock-in mouse model. [*Circ J.*](https://www.ncbi.nlm.nih.gov/pubmed/?term=Dantrolene%2C+a+Therapeutic+Agent+for+Malignant+Hyperthermia%2C+Inhibits+Catecholaminergic+Polymorphic+Ventricular+Tachycardia+in+a+RyR2R2474S%2F%2B+Knock-In+Mouse+Model) 2010 Nov;74(12):2579-84.

2) [Keshavarz M](https://www.ncbi.nlm.nih.gov/pubmed/?term=Keshavarz%20M%5BAuthor%5D&cauthor=true&cauthor_uid=27832686), [Fotouhi M](https://www.ncbi.nlm.nih.gov/pubmed/?term=Fotouhi%20M%5BAuthor%5D&cauthor=true&cauthor_uid=27832686), [Rasti A](https://www.ncbi.nlm.nih.gov/pubmed/?term=Rasti%20A%5BAuthor%5D&cauthor=true&cauthor_uid=27832686). Dantrolene: A Selective Ryanodine Receptor Antagonist, Protects Against Pentylenetetrazole-Induced Seizure in Mice. [Acta *Med Iran*.](https://www.ncbi.nlm.nih.gov/pubmed/?term=Dantrolene%3A+A+Selective+Ryanodine+Receptor+Antagonist%2C+Protects+Against+Pentylenetetrazole-Induced+Seizure+in+Mice) 2016 Sep;54(9):555-561.

3) Sepúlveda M, Gonano LA, Viotti M, Morell M, Blanco P, López Alarcón M, Peroba Ramos I, Bastos Carvalho A, Medei E, Vila Petroff M: Calcium/Calmodulin Protein Kinase II-Dependent Ryanodine Receptor Phosphorylation Mediates Cardiac Contractile Dysfunction Associated With Sepsis. *Crit Care Med*. 2017; 45(4): e399-e408.

4) Maier S, Traeger T, Entleutner M, Westerholt A, Kleist B, Hüser N, Holzmann B,

Stier A, Pfeffer K, Heidecke CD. Cecal ligation and puncture versus colon ascendens stent peritonitis: two distinct animal models for polymicrobial sepsis. *Shock*. 2004; 21:505-11.
